# Diagnostic and Surgical Management of a Thrombosed Distal PICA Aneurysm: A Selected Case Report and Review from a Retrospective Institutional Series

**DOI:** 10.2174/0115734056417096251127114551

**Published:** 2026-07-14

**Authors:** Alejandro Serrano-Rubio, Sharon-Paola Garcia-Trujillo, Brenda-Susana Hernandez-Barrera, Rafael Sánchez-Mata, Ana Margarita-Martínez Cáceres, Ernesto Roldan-Valadez, Edgar Nathal

**Affiliations:** 1 Department of Vascular Neurosurgery, National Institute of Neurology and Neurosurgery “Manuel Velasco Suárez”, 14267, Mexico City, Mexico;; 2 Division of Research, Instituto Nacional de Rehabilitación “Luis Guillermo Ibarra Ibarra”, 14389, Mexico City, Mexico;; 3 I.M. Sechenov First Moscow State Medical University (Sechenov University), Radiology department, 119992, Moscow, Russia

**Keywords:** PICA aneurysm, Posterior inferior cerebellar artery, Giant aneurysm, End-to-end bypass, Thrombosed aneurysm, Posterior circulation, Neurovascular imaging, Cerebral angiography, Microsurgical clipping, MRI in neurosurgery

## Abstract

**Background:**

Distal posterior inferior cerebellar artery (PICA) aneurysms are rare vascular lesions, comprising less than 0.5% of all intracranial aneurysms. When thrombosed, they can mimic neoplastic or infectious processes, leading to delayed diagnosis and potential mismanagement. The complex anatomy of the PICA and its variable relationships with adjacent neurovascular structures further complicate both the diagnostic process and surgical approach. Here, we present a rare case of a thrombosed distal PICA aneurysm, with emphasis on imaging characteristics, differential diagnosis, and surgical considerations.

**Case Presentation:**

A 55-year-old man presented with progressive occipital headache, gait ataxia, and dysmetria. Magnetic resonance imaging (MRI) revealed a 1.6 cm lesion in the left cerebellar hemisphere with central T1 hyperintensity and peripheral enhancement, suggestive of a thrombosed aneurysm or mass. Digital subtraction angiography (DSA) confirmed a partially thrombosed saccular aneurysm at the telovelotonsillar segment of the left PICA. Surgical management involved a left suboccipital craniotomy with microsurgical clipping and thrombectomy. Intraoperative findings confirmed a saccular aneurysm with dense intraluminal thrombus adherent to surrounding cerebellar tissue. Histopathology demonstrated organized thrombus with vascular wall disruption and neovascularization, consistent with chronic aneurysmal changes. Postoperative recovery was uneventful, and the patient was discharged with full neurological function restored. Follow-up imaging showed no residual aneurysm.

**Conclusion:**

Thrombosed distal PICA aneurysms pose a diagnostic challenge due to their atypical radiologic appearance and potential to mimic neoplastic lesions. Recognition of characteristic imaging features, such as peripheral enhancement and central hyperintensity on MRI, can aid early diagnosis and prevent mismanagement. Surgical intervention remains the definitive treatment, requiring careful planning due to the complex vascular territory of the distal PICA. Awareness of this rare entity among radiologists and neurosurgeons is critical to guide accurate diagnosis, avoid misinterpretation, and optimize patient outcomes.

## 1 INTRODUCTION

The posterior inferior cerebellar artery (PICA), the largest branch of the vertebral artery (VA), gives rise to a rare subset of intracranial aneurysms, accounting for only 0.5% of all such lesions [1]. Most PICA aneurysms arise at the VA-PICA junction, although aneurysms involving distal PICA segments are notably less common and more surgically challenging. Management becomes particularly complex when the aneurysm originates at the VA-PICA junction or along the proximal segment, where treatment may necessitate the sacrifice of the parent vessel and potentially compromise critical brainstem and cerebellar perfusion.

The anatomy of the PICA is notably variable, and its aneurysms are typically confined within a narrow neurovascular corridor bordered by the medulla oblongata and the anterolateral cerebellar base [2]. These anatomical intricacies limit both endovascular and open surgical maneuverability, demanding precise case-by-case treatment planning. Given the rarity of these lesions, clinicians must be equipped with multiple management strategies tailored to each patient’s vascular anatomy and symptomatology.

Giant intracranial aneurysms, defined by a diameter exceeding 25 mm, are particularly rare within the PICA territory and represent a unique therapeutic dilemma [3]. Their natural history, initially explored in early studies, such as those by Campos et al., has remained controversial for decades [3]. While some lesions remain clinically silent, most giant aneurysms present with significant mass effect or rupture, prompting urgent intervention. In this setting, both microsurgical and endovascular options have been explored, although the literature suggests that microsurgery may offer more durable results, especially for lesions with thrombotic components or mass effect [4].

The clinical presentation of giant PICA aneurysms is diverse. Patients may develop lower cranial nerve palsies, subarachnoid hemorrhage (SAH), transient ischemic attacks (TIAs), or even infarcts due to thromboembolic events arising from intraluminal thrombus migration [5]. Importantly, incidental diagnosis during neuroimaging for unrelated symptoms is also not uncommon [6]. These varied clinical manifestations further complicate the decision-making process, as neurological deficits can evolve both acutely and progressively.

From a technical perspective, endovascular treatment of large and giant PICA aneurysms remains limited by anatomical constraints and unfavorable recurrence rates following coiling. While intrasaccular embolization offers a minimally invasive approach, it is often ineffective in giant lesions due to the presence of thrombus and irregular aneurysm morphology [7, 8]. In contrast, open microsurgical techniques, although technically demanding, allow direct decompression of the mass effect, resection of the thrombus, and precise vascular reconstruction when indicated.

This article presents a case report derived from a retrospective case series of 11 patients with giant posterior inferior cerebellar artery (PICA) aneurysms, managed at the National Institute of Neurology and Neurosurgery in Mexico city between 2012 and 2024. Among these, a single case demonstrated a partially thrombosed aneurysm in the lateral medullary segment of the PICA, an uncommon and technically demanding presentation. The case was selected for detailed description due to its unique radiological and surgical characteristics. Notably, the correlation between magnetic resonance imaging (MRI) and conventional cerebral angiography played a critical role in both diagnosis and preoperative planning, helping distinguish this vascular lesion from other differential diagnoses, such as lower cranial nerve schwannomas or meningiomas. This case underscores the value of advanced imaging interpretation in neurosurgical decision-making, particularly in rare vascular entities, and aligns with the journal’s focus on the integration of imaging in clinical practice.

## 2 SERIES DESCRIPTION AND PATIENT SELECTION

Between 2012 and 2024, a retrospective case series was conducted at the National Institute of Neurology and Neurosurgery in Mexico city, involving 11 patients with giant posterior inferior cerebellar artery (PICA) aneurysms. All cases included were either ruptured or unruptured aneurysms with a dome diameter ≥25 mm. Clinical presentation, anatomical characteristics, and treatment outcomes were recorded. Among these, only one case demonstrated clear imaging and intraoperative evidence of intradome thrombosis, making it a distinctive and technically complex case. The anatomical and clinical characteristics of the series are summarized in Table **1**, while Table **2** outlines the postoperative outcomes and surgical strategies employed. Below, we present this singular case in detail.

## 3 CASE PRESENTATION

A 55-year-old male with a medical history of systemic hypertension presented to our institution with a 4-month history of progressively worsening neurological symptoms. These included gait lateralization, right upper limb dysmetria, dysphagia, and dysphonia, indicative of posterior fossa involvement and lower cranial nerve dysfunction. The chronicity and progression suggested a space-occupying lesion with mass effect on the medulla or cerebellar structures. The patient had no history of prior neurosurgical or endovascular interventions. No relevant family history or psychosocial factors were identified during the initial evaluation. The clinical timeline was as follows: day 0 marked the onset of symptoms, including headache and instability; day 1 involved the initial CT scan; day 2 included MRI and DSA, which confirmed the aneurysm diagnosis; day 3 was the day of surgical intervention; and by day 5, follow-up imaging showed resolution of symptoms.

Initial evaluation with non-contrast head computed tomography (CT) revealed a posterior fossa lesion located anterior to the cerebellum, without definitive features of calcification or hemorrhage. The mass’s indeterminate appearance prompted further characterization via magnetic resonance imaging (MRI). On T2-weighted sequences, the lesion demonstrated a well-defined contour with heterogeneous signal intensity, displaying both hyperintense and hypointense areas suggestive of internal blood degradation products and possible thrombosis (Fig. **1A** ,**B**). The location in the lateral bulbar cistern and its imaging features raised suspicion for a vascular lesion, but the differential diagnosis still included schwannomas of the lower cranial nerves or a petroclival meningioma.

To clarify the vascular anatomy and confirm the suspected diagnosis, digital subtraction cerebral angiography was performed. In the anteroposterior Towne projection and lateral views, a fusiform aneurysm arising from the lateral medullary segment of the posterior inferior cerebellar artery (PICA) was identified (Fig. **1C**,**D**). The lesion demonstrated partial contrast filling, consistent with a partially thrombosed giant aneurysm, and importantly, preservation of distal PICA flow was noted. These findings confirmed the vascular nature of the lesion and guided the subsequent surgical planning. Given the aneurysm's thrombosed state and distal location, the clinical team considered the prognosis potentially poor without prompt surgical management.

The aneurysm’s morphology, comprising a fusiform shape, a 25 mm dome, and a 7 mm neck, combined with its location in a tight neurovascular corridor and thrombotic component, rendered endovascular options suboptimal. Simple coiling or flow diversion was contraindicated due to the risk of thrombosis propagation or recurrence, and trapping without bypass would have posed a high risk of posterior circulation ischemia. Therefore, the surgical plan prioritized aneurysm resection and restoration of vascular continuity via bypass.

Under general anesthesia, the patient was positioned prone in a Concorde configuration with slight head rotation. A suboccipital craniotomy was performed to access the cerebellomedullary cistern. After durotomy, microsurgical dissection exposed the vertebral artery and proximal PICA, which was dissected free from surrounding arachnoid and cranial nerve structures. The aneurysm was visualized and isolated via proximal and distal temporary clipping. Upon opening the aneurysm dome, a large intradome thrombus was identified and carefully evacuated.

Subsequent aneurysmectomy was performed to relieve the mass effect on adjacent neural structures. Due to the chronic aneurysmal dilation and elongation of the PICA segments, the proximal and distal stumps were sufficiently mobile and well-aligned. This anatomical configuration permitted a tension-free end-to-end anastomosis, executed with standard microvascular techniques. Prior to finalizing the anastomosis, both ends were trimmed with oblique “fish-mouth” cuts to optimize surface area and ensure laminar flow.

To verify bypass patency, fluorescein angiography was used intraoperatively. Injection through a peripheral vein demonstrated real-time transit of contrast across the anastomosis, confirming technical success and adequate flow restoration (Fig. **2A-D**). No intraoperative complications occurred.

The patient’s postoperative course was uneventful. The patient tolerated the surgical procedure without complications and adhered to the postoperative care plan, including imaging and clinical evaluations. He exhibited notable improvement in cranial nerve-related deficits and was discharged on postoperative day 5 in a stable neurological condition. A follow-up MRI and angiogram confirmed resolution of the mass effect and patency of the bypass, with sustained perfusion of the distal PICA territory.

This case illustrates the complexity of diagnosing and managing thrombosed distal PICA aneurysms, which are rare but potentially life-threatening due to their proximity to critical brainstem structures and their often deceptive appearance on initial imaging. The correlation between advanced neuroimaging and intraoperative findings was crucial for establishing an accurate diagnosis and selecting an individualized, anatomically informed surgical approach.

## 4 DISCUSSION

Aneurysms arising from the posterior inferior cerebellar artery (PICA) are relatively rare within the spectrum of intracranial vascular lesions. The majority are located at the vertebral artery-PICA (VA-PICA) junction, which accounts for the higher frequency of proximal aneurysms observed in the literature [9]. When aneurysms occur in the distal portion of the PICA, they account for 0.5% to 6% of the cases [10]. Distal PICA aneurysms, particularly those involving peripheral segments, like the lateral medullary segment, are far less common and represent a unique subset with reported incidence ranging from 0.28% to 1.4% of all cerebral aneurysms [9]. Moreover, most case series that describe PICA aneurysms also include aneurysms involving the VA, underscoring the infreq-uency of isolated distal PICA pathology. Recent reports continue to highlight this rarity, emphasizing the technical and anatomical challenges in diagnosis and treatment planning [11].

The prognosis of giant aneurysms in this location is particularly poor due to their potential for hemorrhage, mass effect, and thromboembolic events. As reported by Drake *et al.* [12], the natural history of untreated giant aneurysms is grim, with mortality rates reaching 66% at 2 years and exceeding 80% at 5 years [2, 13]. These statistics reinforce the need for timely intervention. In this context, preoperative angiographic evaluation is essential, not only for confirming the diagnosis but also for assessing the distal flow of the PICA and determining the status of vascular collaterality [14]. When distal PICA perfusion is maintained, preserving this flow must be a guiding principle in treatment planning. Recent microsurgical case reports, including bypass-assisted resections of dolichoectatic or giant posterior circulation aneurysms, further emphasize the importance of preserving distal flow in high-risk posterior fossa territories [15].

Even in the presence of angiographic collaterality, distal PICA flow should not be compromised, as this artery supplies eloquent areas of the cerebellum and medulla. Ischemia in these territories can lead to significant neurological deficits. Notably, PICA aneurysms differ from anterior circulation aneurysms in that they may rupture at smaller sizes and carry a higher bleeding propensity, which further supports their proactive treatment [16-18]. Recent studies have reinforced this observation, indicating that aneurysms originating from the PICA have a higher rupture risk compared to those in other locations, underscoring the need for vigilant management strategies [19].

The management of giant aneurysms (>25 mm) remains controversial and must be tailored to each case. Both endovascular and microsurgical approaches are viable, each offering distinct advantages and technical limitations [3]. Although the debate between clipping and coiling continues, in cases involving complex anatomy, such as distal PICA aneurysms, especially when associated with thrombosis and mass effect, direct microsurgical inspection remains superior. This approach offers a comprehensive view of the aneurysm’s anatomy, its relationship to neurovascular structures, and the opportunity for bypass when required [20]. Recent literature further emphasizes that in select posterior circulation aneurysms with high morphological complexity, microsurgical approaches provide durable results and better control over thrombosed segments and adjacent perforators [21, 22].

A unique anatomical feature of many PICA aneurysms is the posterior displacement and elongation of the distal PICA segment. This phenomenon results from chronic aneurysmal expansion and, as observed in our case, creates favorable conditions for tension-free end-to-end anastomosis following aneurysmectomy [23]. The end-to-end bypass technique is particularly advantageous in these settings. Compared to end-to-side or side-to-side reconstructions, it provides lower rates of occlusion, better hemodynamic compatibility, and more anatomically faithful vascular repair, especially when the goal is not only to remove the mass effect but also to restore distal circulation and prevent postoperative ischemic complications. Recent innovations in bypass techniques, such as distal PICA displacement with end-to-side anastomosis to the contralateral PICA, further highlight the evolving strategies to manage complex PICA aneurysms [24]. Microsurgery offers several advantages over endovascular treatment in the acute phase of this type of aneurysm. It allows for at least partial blood removal, which can help avoid additional procedures, such as external ventricular drainage and subsequent ventriculoperitoneal shunting, especially in patients with massive intraventricular hemorrhage. Furthermore, in cases with multiple lesions, the source of the hemorrhage can be identified and safely treated, with a lower risk of re-hemorrhage. Endovascular treatment will never be able to offer this important decompressive effect, including hematoma removal [18].

## 5 STUDY LIMITATIONS

This report presents a single case and, as such, may not be broadly generalizable to all distal PICA aneurysms. Although the use of multimodal imaging, including digital subtraction angiography (DSA) and MRI, allowed accurate diagnosis and surgical planning in this case, such comprehensive imaging may not always be available in emergency settings or resource-limited environments.

Interpretation of imaging findings was influenced by the thrombosed component, which may obscure key morphological features, even on advanced sequences. Additionally, while the histopathological analysis confirmed aneurysmal nature and thrombus organization, immunohistochemistry and molecular characterization of the aneurysm wall were not pursued, limiting insights into pathophysiologic mechanisms.

Future studies involving larger case series and integration of vessel wall imaging, genetic markers, and long-term follow-up could provide a deeper understanding of risk factors, recurrence potential, and optimal treatment strategies for thrombosed PICA aneurysms.

## CONCLUSION

Thrombosed giant aneurysms of the distal posterior inferior cerebellar artery are rare, anatomically complex, and clinically significant lesions. Successful management relies on timely diagnosis, high-resolution imaging, and tailored microsurgical strategies. In selected cases where distal flow is preserved, aneurysmectomy with end-to-end bypass reconstruction offers a definitive and effective solution. Our case reinforces the value of integrating advanced neuroimaging with anatomical and surgical insight to optimize outcomes in these high-risk patients.

## Figures and Tables

**Fig. (1):**
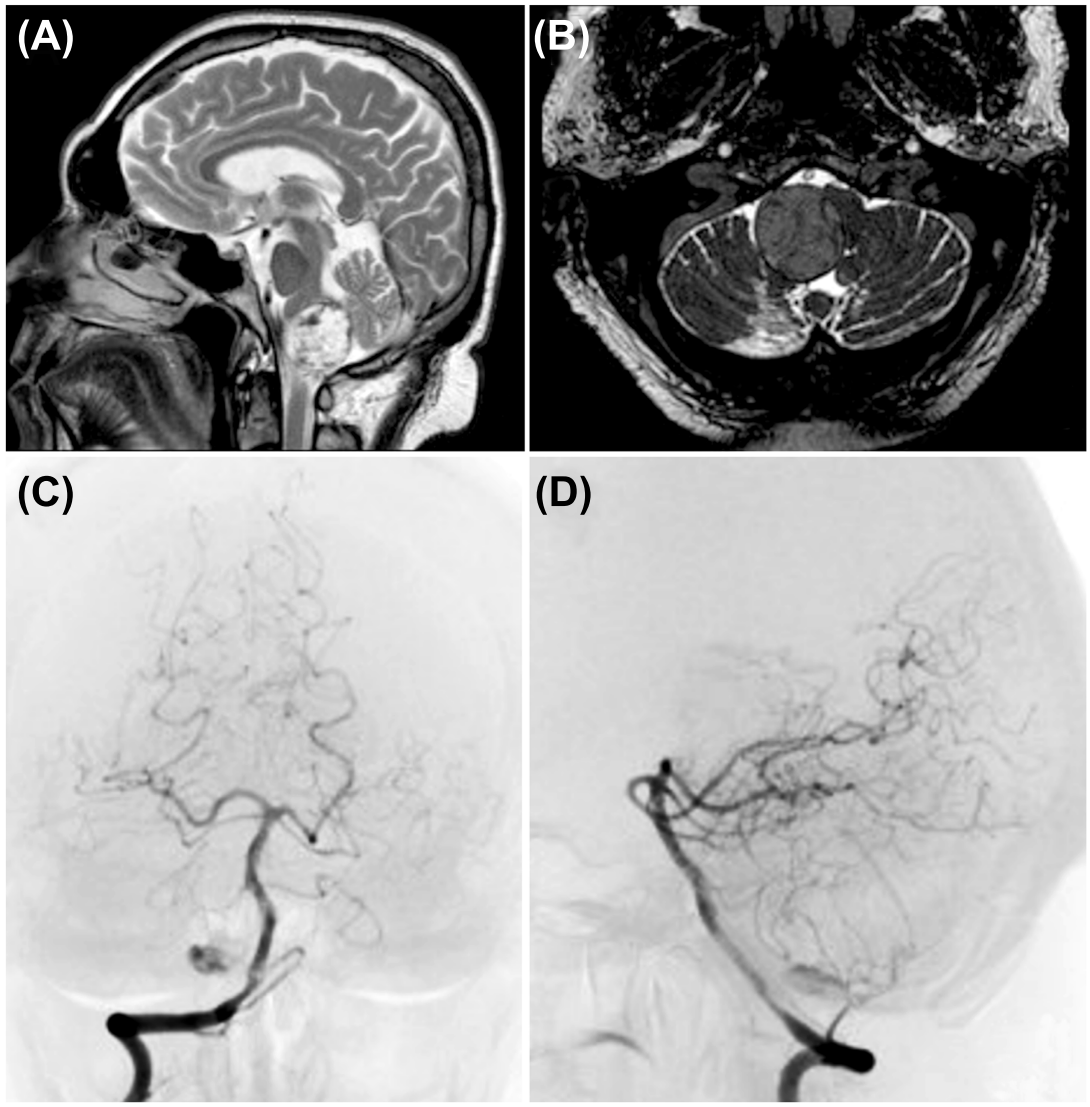
Preoperative MRI and cerebral angiography findings. (**A**,**B**) Axial and sagittal T2-weighted MRI showing a well-defined lesion located ventral to the cerebellum, within the lateral bulbar cistern, with heterogeneous signal suggesting partial thrombosis. (**C**) Digital subtraction angiography, anteroposterior Towne projection, revealing a fusiform aneurysm arising from the lateral medullary segment of the PICA. (**D**) Lateral projection angiography demonstrating partial contrast filling of the aneurysm dome, with preserved distal PICA flow.

**Fig. (2):**
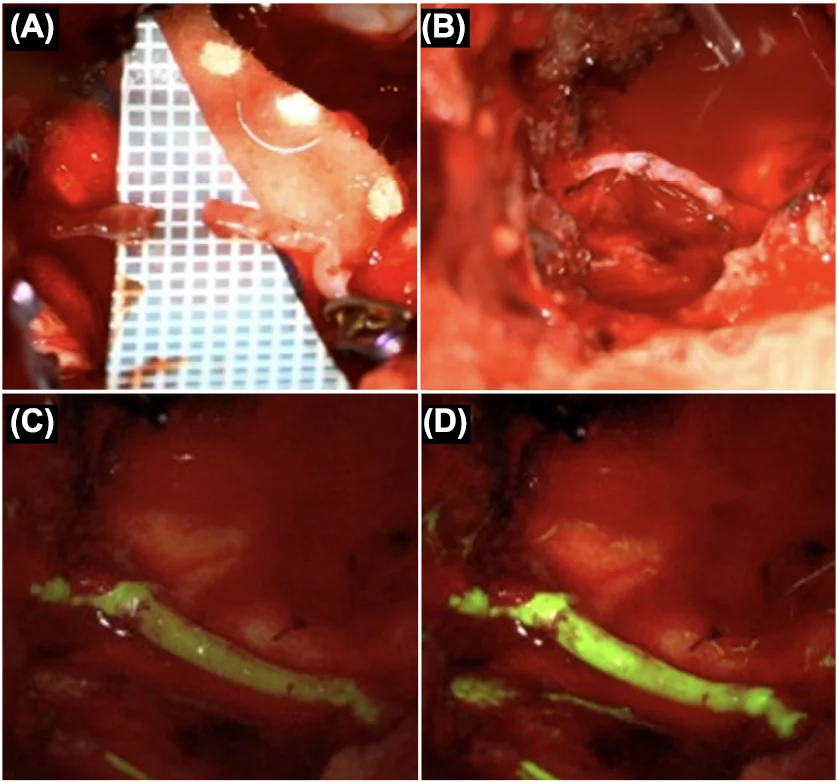
Intraoperative views and fluorescein videoangiography. **A)** Intraoperative photo showing proximal and distal segments of the PICA, aligned to permit tension-free end-to-end bypass anastomosis. **B)** High-magnification microphotograph illustrating the completed end-to-end PICA bypass. **C)** Intraoperative view of the bypass during early fluorescein injection, confirming flow initiation. **D)** Later phase of fluorescein angiography, demonstrating robust flow through the reconstructed PICA bypass.

**Table 1:** Anatomical and clinical characteristics of patients with giant PICA aneurysms (n = 11). This table summarizes the anatomical measurements and clinical presentations of 11 patients diagnosed with giant aneurysms of the posterior inferior cerebellar artery (PICA) treated at the National Institute of Neurology and Neurosurgery, Mexico city, between 2012 and 2024. Dome and neck dimensions were obtained from neuroimaging studies. Thrombus presence was confirmed by preoperative imaging or intraoperative findings. The table also documents the presence of cranial nerve and motor symptoms, as well as any associated arteriovenous malformations (AVMs).

**Patient**	**Sex**	**Dome Size (mm)**	**Neck Size (mm)**	**The Presence of a Thrombus**	**Cranial Nerve Symptoms**	**Motor Symptoms**	**The Presence of AVM**
1	M	12 × 6	6	No	No	No	No
2	F	–	–	No	Yes	Yes	Yes
3	M	25	2	**Yes (80%)**	Yes	No	No
4	F	4.8 × 3.6	3.6	No	Yes	No	No
5	M	12	2.89	No	Yes	Yes	Yes
6	F	6 × 1.5	1.5	No	Yes	No	No
7	F	7 × 3	3	No	Yes	No	No
8	F	7.49 × 5.76	2	No	No	No	No
9	M	4.3	5	No	No	No	No
10	F	5	2	No	No	No	No
11	M	25	7	**Yes**	No	No	No

**Abbreviations**: M, male; F, female; PICA, posterior inferior cerebellar artery; AVM, arteriovenous malformation. **Note**: Dome sizes presented as length × width where available.

**Table 2:** Functional outcomes and surgical techniques in patients with giant PICA aneurysms (n = 11). This table presents functional outcomes, distal PICA flow status, and surgical techniques used in 11 patients treated for giant PICA aneurysms. Functional status was measured using the modified Rankin Scale (mRS) before and after surgery. All patients demonstrated preserved distal flow on follow-up angiography. Bypass procedures were performed in cases where anatomical reconstruction was required following aneurysm resection.

**Patient**	**Pre-op Rankin Score**	**Post-op Rankin Score**	**Distal PICA flow Preserved**	**Surgical Technique**
1	1	0	Yes	Clipping
2	2	2	Yes	Clipping
3	3	1	Yes	Clipping + bypass
4	4	2	Yes	Clipping
5	3	2	Yes	Clipping + bypass
6	4	2	Yes	Clipping
7	5	5	Yes	Clipping
8	1	0	Yes	Clipping
9	0	0	Yes	Clipping
10	1	0	Yes	Clipping
11	1	0	Yes	Clipping + bypass

**Abbreviations**: PICA, posterior inferior cerebellar artery; mRS, modified Rankin scale.

## Data Availability

Not applicable.

## LIST OF ABBREVIATIONS

PICA: Posterior inferior cerebellar artery
VA: Vertebral artery
MRI: Magnetic resonance imaging
CT: Computed tomography
DSA: Digital subtraction angiography
SAH: Subarachnoid hemorrhage
mRS: Modified Rankin Scale
AP: Anteroposterior
AICA: Anterior inferior cerebellar artery

## STANDARDS OF REPORTING

CARE guidelines were followed.
